# Behavioral and metabolic profiles in an aged, humanized *APP/APOE* mouse model of Alzheimer’s Disease risk

**DOI:** 10.1002/alz.091716

**Published:** 2025-01-03

**Authors:** John W. McLean, Avnish Bhattrai, Roberta Diaz Brinton

**Affiliations:** ^1^ University of Arizona, Tucson, AZ USA

## Abstract

**Background:**

Research into Alzheimer’s Disease (AD) pathomechanisms frequently utilizes animal models with dominant mutations; however, the vast majority (>95%) of AD cases are idiopathic. Animal models with AD risk factors represent an approach with potentially greater translational validity. The predominant genetic risk factor for AD is the Apolipoprotein E *ε4 (APOE4)* polymorphism, with *APOE4* homozygosity conferring approximately 15‐fold higher risk relative to the normative *APOE3/3* genotype. Additionally, women are nearly twice as likely to develop AD. To address the translational validity of a risk factor model approach for AD research, we investigated behavioral and metabolic differences in a novel mouse model with homozygous expression of humanized (h) amyloid precursor protein (h*APP*), encoding the precursor of the major protein in amyloid plaques, and replacement of murine *Apoe* with humanized h*APOE3* or h*APOE4*.

**Method:**

Aged (22‐24 months) h*APP*/*APOE3* and h*APP*/*APOE4* mice underwent open field (OF), novel object recognition (NOR), EchoMRI body composition analysis, fasting blood glucose (FBG) and ketone body (FKB) determinations. Data were analyzed by 2‐way ANOVA for effects of biological sex and *APOE* genotype followed by post‐hoc *t*‐tests and considered significant at p<0.05.

**Result:**

Female mice traveled significantly farther and for a greater percentage of time during both OF and NOR, and interacted more with both familiar and novel objects during NOR. There were no group differences in either thigmotaxis or novelty recognition. EchoMRI body composition analysis revealed significant weight reduction in both male and female *APP*/*APOE4* mice. While weight loss included a decline in both lean and adipose mass, greater loss of adipose tissue in *APP/APOE4* mice resulted in lower body fat percentage and was the major contributor to weight loss.

**Conclusion:**

Metabolically, female mice had lower FBG and higher FKB relative to males. The sex difference was greater in *APP/APOE4* females, which had the lowest FBG and highest FKB. The h*APOE4*‐driven reduction in body weight, especially in adipose tissue, further indicates a metabolic consequence in mice expressing the AD risk gene. Collectively, this program of research contributes to the determination of the translational validity of the humanized *APOE* and *APP* mouse model for preclinical target identification and therapeutic development for AD.

